# “I Put Her in the Baby Stroller and Left”: The Escape Route From Violence to a Domestic Violence Shelter for Mothers and Children

**DOI:** 10.1177/10778012241251971

**Published:** 2024-05-02

**Authors:** Sara Thunberg, Linda Arnell

**Affiliations:** 1 School of Behavioural, Social and Legal Sciences, 6233 Örebro University, Örebro, Sweden; 2 Department of Social Work, 8075 Umeå University, Umeå, Sweden

**Keywords:** children, domestic violence shelter, escape, intimate partner violence, leaving process

## Abstract

Little is known about mothers’ and children's escape from violence and its aftermath when living in secure accommodation, especially with regard to children. The aim is to investigate mothers’ experiences of their escape, and their considerations regarding the well-being of their young children before or during their escape, based on 14 interviews. Using a narrative thematic analysis, the results show that the escape was often planned, but that the planning horizon varies. In many cases, the mothers’ social network served as a stepping-stone during the escape, before they continued by moving to a domestic violence shelter (DVS). Implications for policy and practice are offered.

## Introduction

Although it is well known that many children experience domestic violence during their childhood, the issue continues to exist. This results in women and children having to flee their homes to live in domestic violence shelters (DVSs) for varying amounts of time. The violence that children experience ranges from witnessing violence between parents, violence toward a sibling, violence toward the child directly, or combinations of all of these (Gilbert et al., 2009). According to Fernández-González et al. (2018), as many as 80–90% of the children living in DVSs have witnessed the violence, while 50% have been abused themselves. In Sweden, where the present study was conducted, 40% of all children have experienced abuse from an adult (Jernbro et al., 2023), 10–15% have witnessed physical violence between their parents, and 30% have witnessed verbal conflicts (Annerbäck et al., 2010; Cater et al., 2015; Jernbro et al., 2023). Among children who have experienced violence against their mother, 77% were in the same room and 95% were at home on at least one occasion when the mother was abused (Almqvist & Broberg, 2014).

Experiencing violence at home while growing up can result in various consequences for children. It is a serious risk factor for developing physical, mental, and social problems, including symptoms of posttraumatic stress, anxiety, and nonsuicidal self-harm (Annerbäck et al., 2012; Cater et al., 2014, 2015; Gilbert et al., 2009; Holt et al., 2008; Jernbro et al., 2023; McTavish et al., 2016). These are consequences that can follow the child into adulthood. Among children who are exposed to more than one form of violence, the risks are particularly great, and research shows that 25% of these children have attempted suicide (Jernbro et al., 2023). This means that early exposure to violence, either as a witness or the direct target, can have serious short- and long-term consequences. How and to what extent children react can vary (Howell et al., 2010; Levendosky et al., 2002), but in order for them to be able to live with these experiences, it is crucial that they receive safe accommodation and the support they need (Hauge & Kiamanesh, 2020; Theobald et al., 2021).

One way of ending the violence is for the parents to end their relationship. However, in relationships where violence occurs, it is rarely that easy. Instead, the nonviolent parent, often the mother, needs to flee the violence together with the children. Having children might, however, act as both a hindrance and an enabler in terms of leaving the violent relationship. In cases where it is a hindrance, this often comes down to fear, often due to threats from the perpetrator, that she will lose the children if she leaves, or that she wants to keep the family together (Rhodes et al., 2010; Zink et al., 2003). Expressed differently, fleeing into the unknown and feelings of fear and loss of control might be viewed as a worse alternative than staying (Hydén, 1999). In other cases, where having children works as an enabler for leaving the violent relationship, the mothers’ arguments often come down to protecting their children from the violence because it might affect them negatively (Rhodes et al., 2010; Zink et al., 2003).

After leaving a violent relationship, it is common to end up in a DVS. These are often seen as a place of safety from the perspective of both mothers and children, and safety is also one of the factors that mothers value the most about a DVS (Arnell & Thunberg, 2023; Chanmugam, 2011; Jonker et al., 2014; Vass & Haj-Yahia, 2020). Mothers also value the fact that the shelters can provide health care, legal assistance, and support for children (Sullivan & Virden, 2017). In addition, Thunberg et al. (2022) show that children often feel safe at the shelter, and that the play areas and activities offered by the DVSs are of great value. However, their review also showed that if there is a lack of security at the DVS and children do not feel safe, this affects other aspects of the children's experience, such as the possibility to participate in leisure activities, go to school, and make new friends (Thunberg et al., 2022). Similarly, Arnell and Thunberg (2023) show that activities and social relationships are important for children in a DVS, but they also highlight the importance of a child-friendly environment and support for children during their stay.

In addition, when children move to a DVS, they leave behind their home, friends, family, school, and possible recreational activities and enter an unfamiliar situation (Chanmugam, 2011; Øverlien et al., 2009; Vass & Haj-Yahia, 2021). Children may also try to figure out why they are leaving their home, where they are going, and what they should take with them (Vass and Haj-Yahia (2021). Vass and Haj-Yahia (2023) also show that experiences of exposure to violence against their mother have an ongoing impact on children, particularly in the shelter setting. The lives of many children who experience domestic violence are also affected by frequent moves. This includes not only the initial move to the shelter, but also moving between different secure accommodations, which leads to frustration, anger, and a sense of helplessness and insecurity (Chanmugam, 2011; Vass & Haj-Yahia, 2021).

The escape route that mothers and children take from a violent home to a DVS is rarely discussed in research. However, the research on escaping violence for shelter elsewhere is growing, and it shows, for example, that the children rarely know in advance that they are leaving their homes and have little time to prepare (Øverlien et al., 2009; Vass & Haj-Yahia, 2021). Instead, the move is experienced by the children as an unexpected, sudden event that leads to uncertainty about what is happening (Vass & Haj-Yahia, 2021; Weisz et al., 2005). When children explain the reasons for leaving, they mention fear that their mother will be injured or die, or the need to gain some freedom or space, and to teach the father a lesson (Vass & Haj-Yahia, 2021). However, escaping the violence is often only the beginning of a long process (Hydén, 1999).

There are approximately 280 DVSs in Sweden. The latest survey conducted by the National Board of Health and Welfare (2020) shows that 54% of the shelters are run by nonprofit organizations, 37% by private companies, and 9% by various municipalities. Each year, as many as 6,500 adults and 6,200 children, aged 0–17, stay at a DVS for at least one night (The National Board of Health and Welfare, 2020). This is a 38% increase since the previous survey in 2012. Furthermore, 78% of the DVSs in Sweden report having to turn away people seeking assistance (The National Board of Health and Welfare, 2020). This suggests that the number of people in need of protection is considerably larger than the number of places available. This is problematic, as it may leave mothers and children in dire need to escape from violence in the position of having to look out for themselves.

In summary, there is a growing body of research on children's experiences of violence and DVSs. Still, research on the physical escape from a violent home to a DVS is sparse, pointing to a need for more research that takes into account different perspectives, contexts, and welfare systems. To better support mothers and children when leaving violent homes, knowledge and understanding of the process of escaping violence is of great importance. The aim of this study is therefore to investigate mothers’ experiences of their escape, and their considerations regarding their young children's well-being before or during the escape. The focus is thus not on the whole process of leaving, but only on the (physical) escape from violence.

### Research Questions

1. How do mothers describe their escape and move to a DVS?
2. What considerations do they make in relation to their children's well-being?

## Theoretical Approach

The process of leaving violence, according to Enander and Holmberg (2008), includes the process of breaking up, the process of becoming free, and the process of understanding, although they do not necessarily occur in that order. The process of breaking up, according to Holmberg and Enander (2005), covers action—in this case the escape route from violence to a DVS—and the turning points which precede it. It can thus be understood as part of a longer process of leaving and can be understood in relation to the theory of the exit process developed by Fuchs Ebaugh (1988), which is discussed in terms of turning points.

Turning points are described by Fuchs Ebaugh as dramatic and sudden changes, even though sometimes they appear gradual. A turning point can be related to various factors, such as an extreme or extraordinary event, the realization that it is enough, that time is running out, and that the alternatives have run out, or simply that there are no more excuses. According to Holmberg and Enander (2005), danger to life or the danger to someone else, for example, a child, is often an important turning point for abused women. Fuchs Ebaugh (1988) also discusses the importance of the reactions from one's social network, especially during the time leading up to a turning point. However, according to Holmberg and Enander (2005), the network does not seem to be as important, which indicates that previous research is not conclusive regarding the role of social networks in leaving a violent relationship.

## Method

This article is based on a project called *Article 19: What sheltered housing means for abused children*. The project consists of two substudies focusing on children's experiences of: (1) living at a DVS with their mother and, potentially, siblings and (2) being denied a place at a DVS for any reason. The data consists of interviews with children aged 7–17 years and mothers of children aged 0–6 years. For this article, data from both substudies has been used, and the focus is on the process of escaping from violence as described by the mothers of the youngest children. This choice of focus is based on the analysis of the interviews, as it became clear that the escape process affects the stay at the shelter or other types of secure accommodation. For this reason, the experiences of the shelter stay are reported in other articles, while this article focuses solely on the process of escaping to a DVS or other secure accommodation. Similarly, the article only focuses on the interviews with the mothers (i.e., the youngest children) because of the importance they attached to the escape and their detailed narratives of the process of leaving. The experiences of the children aged 7 years and above are reported in another article.

### Procedure

This article uses semistructured interviews with 14 mothers, which were conducted between February 2021 and November 2022. They were between 27 and 47 years old, with an average age of 35.6 years. The mothers had between one and three children living with them. All but one of the mothers had prior experience of staying at DVSs. Of these, eight were still living at the shelter at the time of the interview, and most had a plan for when and how they would move out of the shelter. The shelter stays ranged from a couple of months to 3 years, with several of the mothers having moved between shelters and thus having experienced multiple shelter stays during the period.

The interviews were semistructured (Brinkman, 2014), allowing the participants to tell their stories in their own words with little interference from the researchers. Interview guides were used, but the order of the questions was varied in order to follow the participants’ stories about their experiences. These guides were also the primary research tool. The interview guides, and therefore the interviews, were structured around the phases introduced by Rösare (2015)—the first day, the first week, the ongoing life, the move from the shelter or secure accommodation, and the time after leaving. These phases are described by Rösare (2015) as important for understanding the life situation of mothers and children leaving violent homes in relation to staying at a DVS. However, Rösare does not discuss the escape from the violence and its potential impact on the well-being of children or mothers.

The mothers were introduced to these phases due to their use as a structure for the interviews. The phases do not focus specifically on the process of escape but, as part of the interview, the mothers were asked to talk about what their lives were like before they escaped the violence and the situation at the point when they left. Depending on the mothers’ narratives, follow-up questions were asked to elicit more in-depth answers or clarifications. While analyzing the narratives, we found that the process of leaving, and more specifically the escape from violence, is important in the mothers’ narratives and is described as influencing the shelter stays for their children. As the research is sparse, and knowledge about the process of leaving domestic violence is important, this article focuses on what happens before the five phases described by Rösare (2015). The interviews were audio-recorded and transcribed by the authors. No reimbursement was offered for participation.

The length of the interviews varied, but they lasted approximately 60 min. They took place in various locations; for example, at a shelter, in the mothers’ homes, over the telephone, or via Zoom, depending on the participants’ preferences. They were recruited with the help of staff at DVSs in Sweden. The staff informed the mothers staying at the shelter about the study, as well as those who had stayed there during the previous 3 years, and information about the project was also posted on the university's webpage. The mothers who then decided that they wanted to participate either consented to the staff informing the researchers, or contacted the researcher directly themselves.

### Ethics

Before the study began, ethical approval was obtained from the Swedish Ethical Review Authority (Dnr: 2020:04561, 2021-03242, 2021-06928-02). The sensitivity inherent in the subject has influenced everything within the project, from planning to execution and presenting the results. As described, the mothers received information about the study from shelter staff. They could also read about the project on the university's website. Before each interview, the mothers were also given the information by the researcher conducting the interview, and they had an opportunity to ask questions about the project and their participation. Informed consent was then obtained before the interviews began, and they were informed that they could withdraw their consent at any time. In the article, personal names, names of organizations, and places have been replaced with pseudonyms or omitted to protect the identity of the participants. To minimize the risk of retraumatization, the interviewers were attentive to individual situations and the well-being of the mothers. Information about support services was also handed out to all participants. In addition, the shelter staff also served as a source of support, helping to meet the mothers’ need for support, security, and well-being.

### Data Analysis

As mentioned earlier, the phases presented by Rösare (2015) provided the foundation for the interviews. Still, within the broader themes, we conducted a thematic analysis focusing on what the women described as important during each phase, including the time of the escape, for their young children. When conducting this thematic analysis, a combination of narrative thematic analysis (Riessman, 2008) and Braun and Clarke's (2006) six steps for thematic analysis were used. Starting with narrative thematic analysis, our focus was on understanding parts of the material in relation to the narrative as a whole, making it a back-and-forth process. This perspective provides the foundation for the analysis, which will be demonstrated by the presentation of quite long quotes from the participants. These substantial quotes are intended to give a broad picture of the narratives, rather than short sentences that can be misinterpreted if not placed within their context. The quotes were translated from Swedish to English by the authors, and then reviewed and revised by a native English speaker who had access to the original quotes were for comparison. This was done in order to make the translation as accurate as possible. The six steps of thematic analysis are used as a complement to the narrative analysis as a way of structuring the analytical process. To strengthen the quality of the analysis, each of the authors coded the interviews and constructed the themes separately. The analysis began with (1) separately familiarizing ourselves with the data by transcribing the interviews, reading the transcripts multiple times, and taking notes. Then (2), based on the notes and our reading, we separately generated our initial codes, followed (3) by the authors’ separate constructions of the themes regarding the process of leaving, and more specifically the escape route from a violent home to a DVS. At this point, we reviewed and discussed (4) our separate coding and thematizations, which led to some revisions. Also, during this process, the subthemes were clarified, and we took the next step: using the theories on the leaving process to develop the analysis. The theoretical framework of the leaving process and turning points made it possible to analyze the narratives in more depth. This was followed by (5), naming the themes and subthemes in a way that represents both the content of each theme and how the themes interact with each other. Finally, (6) we wrote our report of the findings, presented below, which described the escape from domestic violence and the move to secure accommodation. The two authors were equally involved in the analysis process, while the first author has taken the lead in reporting the findings.

## Findings

The findings consist of two themes: *Timeframe—the planning horizon during the escape* and *Support*—*informal and formal help during the escape*, each containing two subthemes. Both themes are interlinked in relation to the escape, with timeframe focusing on the actual escape from the violence, and support focusing on what kinds of help (both formal and informal) that the mothers received while escaping. These themes are closely interlinked, as the timing of the escape and the support received both affect the leaving process, including the ability to minimize the risk of violence during the escape, the emotional support and motivation to leave, and the obtaining of formal assistance and protection from the violence in the form of being placed at a DVS (see Table 1). The analysis presented below shows that the escape from violence takes different routes, with the help of various people, which needs to be addressed and understood as influencing how both mothers and children experience life at the shelter and the support they receive.

### Timeframe—The Planning Horizon During the Escape

This theme consists of two subthemes related to how the process of leaving looked in relation to time. The subthemes were closely interlinked with each other because, in most cases, the mothers in our study had some time to plan their escape. However, the planning horizon varied, and some had only a very short time. Although the planning horizon varied, a longer timeframe helped the mothers minimize the violence and leave on their own terms, although this was influenced by the urgency of the situation.

#### Fleeing in Panic

The first subtheme focused on those who had to escape instantly, with just what they had with them at the time, and those who had up to a couple of hours. Four mothers described being forced to flee in panic with more or less only the clothes they were wearing, due to the risk to themselves and their children. Erika described it like this:

That night, it was another rape and then another on Sunday and I felt… I felt such fear and panic then, I felt “am I not going to be left alone? Even if we break up, then I don’t know where this is going to end.” So, it ended with me fleeing from him in panic. I ran from him, barefoot; I barely had any clothes on. I called the police, and it became a big police operation to arrest him so that we could go in and get [my son]. Then we ended up in this shelter for women. (Erika)

Erika had been subjected, among other things, to sexual violence. She explained that she had had enough, and when her partner said that they should break up, she agreed. However, she was raped yet again, and she felt that it would never end. She therefore fled in panic and called the police for help to get her son.

Helena similarly described how she had tried to leave her violent partner, but that he did not accept that, and withheld the children from her. Because of this, she contacted social services in her acute situation to receive help. Social services made the assessment that they needed a shelter and put her and her children in a taxi to drive to a shelter. In Amelia's case, both social services and the police came to her house due to the violence. Amelia was removed from the house, but it was uncertain whether her son would be able to accompany her, as social services needed confirmation that he could be removed from the home immediately. In other words, there was a risk that Amelia would have to leave her son behind if she was to go with social services to sheltered accommodation. Celine similarly described an acute situation, where she had to flee immediately:

He said that we should put away the butchering knives from last summer because otherwise he didn’t know what he might do with them. At that point, I feared for my life. Then he said “now you will go upstairs, and you won’t come down until I’ve left for work tomorrow” […] Then I called the domestic violence helpline and told them what had happened. Then they said: “You have to get out of there immediately.” At that point I got scared and called the police. The police didn’t have a car to come and collect us, so we had to walk. It was dark outside; it was the middle of the night. We had to walk a long way. (Celine)

Celine's description clearly shows that there was a risk to her own and her children's lives, based on what her husband told her. However, there was a higher risk in fleeing immediately, due to his statement. Therefore, she agreed to being upstairs with the children until they could escape. Then, when her husband left home later that night, they had an opportunity to escape. At that point, she took the children and walked away to protect her own and her children's lives.

This section shows that, in some cases, mothers and children do not have much choice other than to escape the violence as soon as possible, generally with only the personal belongings they have on them. This means that, regardless of whether they are given a place at a DVS or are forced to resolve the situation in another way, they might need practical help to retrieve belongings from home, if that is possible for security reasons, or to receive help in buying new things, such as clothes and toys for the children. Lina, for example, described her and her children's escape:

I told the children that we were going to [the city] where [friend of the mother] lives the next day. “Here's a bag for each of you, pack what you want to bring.” And then when we arrived in the city, [name of the younger daughter] became really, really sad and I said: “What's the matter?” “I have no toys with me” [imitating the daughter]. Then I asked: “Didn’t you pack your toys in the bag you’ve got?” It turned out that, oh, she had this big stuffed elephant and that was all she could fit in her bag. So, that was all she had with her. She was: “All my other toys, where are they?” Poor thing. (Lina)

Based on this quote, it is clear that fleeing home in panic and being able to only bring a few things can lead to strong reactions, when the realization hits that you might not be able to return home ever again. Fleeing in panic can also mean less time to explain to the children what is going on, especially—as in this case—when the children are very young. The process of escape can therefore affect the arrival at the refuge, whether it is a DVS or somewhere else, such as the home of a family member or friend. It might be only after arriving that they first realize what just happened and that they have fled the violence, and that they have also left most of their belongings behind. This can be difficult to cope with, especially for the children, as some of their toys may be objects that made them feel safe and gave them comfort. In many cases, the children were also unaware of what was happening and what was going to happen, meaning that everything came as a shock to them.

#### Planned Escape

Compared to the previous subtheme, this subtheme focused on those who had a slightly longer time to plan their escape. For example, those who had a couple of days or weeks to plan when it would be best to leave, meaning the least risk for the mothers and their children. A common factor among those who had a bit more time was that they had some general idea of how to escape beforehand, meaning that they knew which steps they should take to protect themselves and their children. They were able, for example, to hide away their identification cards, money, diapers, and so on, so they could flee as soon as they had an opportunity. In some instances, the women described having made plans beforehand for how they would escape, but that these plans could have been put in place within a short timeframe. Sofia explained that she had been thinking about leaving for a year or so, and that she had asked for help a few months before the escape. However, it was a life-threatening act of violence that led to the escape, described by Sofia like this:

A few months before, I had been in contact with [a support group] … “you have to do this and that, for the sake of the children.” And I had that in the back of my mind, this plan. So, I had made a plan for myself, you have to do this if something happens. So, I… let's see. So, I took my wallet, passport identification. Money, my purse, keys, you know like that. (Sofia)

Sofia described how the escape was planned but, in the end, it had to be put in place within a short timeframe. Still, she managed to plan the escape for a time when her partner was away at work, to make sure that she managed to take all of her own and her child's essentials with them when they fled.

Jacqueline also described how a specific act of violence forced her to leave. In her case, the violence was also turned toward their daughter, who was just a few months old. She described how her partner held their baby's face under running water, tried to strangle her, and hit her.

He couldn’t control himself or stop once he’d started hitting. He didn’t stop until he became tired, sort of. Then, when my daughter was around one and a half month, he started beating her too… So, when she was just over four months old, I’d had enough when he hit me and pulled my hair and I yelled and fought so she woke up. Then I felt that she shouldn’t grow up with this. It's enough now. So, the day after, I took her in the baby stroller and left the house and took myself to a train and just left […] I’d been out all day in the yard clearing weeds so I wouldn’t get stuck inside the house with him and so that she [the daughter] wouldn’t get stuck inside the house with him. I sort of sneaked stuff out, like one diaper at a time and I hid it under the reclining part of the stroller, hid my wallet, the phone, then, turned off the sound. (Jacqueline)

It was the final situation described above that became the turning point, but Jacqueline talked about how she had planned the escape beforehand. She described how she used the stroller to hide away personal belongings, and essentials for her daughter. These narratives show that the process of leaving can be planned a long time ahead, but that the escape itself may be sudden. However, the narratives show that, even when they had just a little time, the mothers planned their escape in various ways. Packing essential things for their children and themselves is one example, while another was given by Rebecka. She described how she took the time to talk to her oldest daughter about what was going to happen.

My daughter, my oldest daughter, she's wonderful. Understands a lot. She wasn’t like other children, instead she understood what was happening. There was no point in hiding anything from her, instead I’m very straightforward with my children when it comes to something that will affect their lives forever. (Rebecka)

Rebecka emphasized that she thought it was important to tell her children about what would happen—about the escape—especially because it affected her children's lives so completely. The example given above also shows that the mothers planned their escape in various ways and that their children and their wellbeing were included in these plans. Sometimes, the planning takes place at short notice and in other cases it continues for months.

In summary, this subtheme showed how the mothers planned their escape from the violence. Here, it is important to reflect upon what goes into the word *planning* as the timeframes differ in relation to the acuteness of the situation, and thus whether the planning takes place over a few hours or, as in Sofie's case, a few months. Even though the stories presented in this section involved a longer planning horizon than the previous section, it was still not very long. Instead, the women often had some general plan so that, if they had to escape, they knew what steps to take. Still, what made the mothers start their planning often came down to an extreme or extraordinary event and the protection of their children. In the quote from Jacqueline above, for example, it was the fact that she did not want her daughter growing up with violence.

### Support—Formal or Informal Help During the Escape

Interlinked with the previous theme was the importance of support. This theme also consists of two subthemes. These subthemes are related to how the process of escape looked in relation to support; more specifically, the way in which relatives, the police, and social services were of help during the escape. Members of the social network provided informal emotional support, and they sometimes helped the mother and children physically move from the home to a secure accommodation. Formal support, such as that provided by the social services and the police, included guidance, support and protection during the escape, and assistance in obtaining a place at a DVS.

#### Relatives and Friends as a Stopover on the Route to a DVS

In the narratives, a personal social network was highlighted as an important aspect for the escape route. In these cases, the mothers and their children first fled to a relative, often the child's grandmother, or a close friend of the mother. Their homes became a stopover between the violent home from which they were fleeing and the DVS. It was also from here that direct contact was made with shelters, the social services, or the police, in cases where this contact had not already been made during the acute phase. Using the help of relatives and friends was most common among those who had some time to plan their escape, but it was also described as a solution when fleeing in panic. Accordingly, staying with a relative or friend was described as a temporary solution. It was a way to stay safe until a place became available at the shelter. Cecilia explained:

I actually did so, I took as much stuff as possible, he [the father] was asleep so he didn’t notice anything anyway, and I took the kids with me and then my mother came and collected me and drove me to my grandmother and grandfather, and I thought I would stay there. As soon as I arrived, I called the social services. And then I told them that… I called them and then I said that I need… that “I’m being abused at home,” something like that. “I need to talk to someone,” and she took my details and connected me. Then, approximately three hours later, I was on my way to a shelter. (Cecilia)

Cecilia had asked her mother for help beforehand, and in that sense their escape was planned beforehand. However, the stopover at the grandparents’ place was brief, just a few hours, and she described the escape to the shelter as happening faster than she had expected.

The length of the stopover with relatives or friends varied, and could be between a couple of hours and up to a week, as the quotes below, from Jaqueline and Rebecka, show:

Approximately a week we lived with a relative, and then we received a place at a shelter. (Jaqueline)

Since there was no space in the domestic violence shelter the first week, we had to stay with my sister. (Rebecka)

Jaqueline and Rebecka both described the stopover as a temporary solution, staying there while they waited for a place at the DVS. However, the stopover with a friend or relative was not just for those who were waiting for a placement at a DVS. It could also be a final stop, if a placement at a DVS was turned down. As an example, Lina was asked by social services to leave with her children for a DVS but, not knowing what a shelter was and afraid of what might happen, she instead took her children and escaped to a friend's place in a different city to get away from her violent partner. The initial idea was to stay there for a couple of days, and then return home, but that was not possible. She explained:

Then I started to talk about wanting to go home and [social services] said no, you can’t do that. You can stay there another week, and if that's not possible, then we’ll organize somewhere for you to stay. And it was fine to stay where we were […] We stayed with [my friend] for almost two months. And it was… I… It took a while before I accepted the situation. (Lina)

Thus, fleeing to a relative or friend could be a way of taking the children to a place that is somewhat familiar, making the transition easier. It could also, as in Lina's case, be a way of avoiding a placement at a DVS, and instead trying to retain some freedom by moving to a friend's place and a familiar environment in a different city. However, according to the mothers’ descriptions, this does not mean that the children knew what was going to happen or why they were leaving their homes. Such a move could be interpreted as going to a known environment, but it was still an unknown situation without control. Once again, this could be interpreted as a way of protecting the children from the unknown, as well as for the mother to receive support for her next step in the process of leaving, protecting the children, and having the best interests of their children in mind when trying to live a life without violence.

#### Help From the Police and Social Services When Fleeing to a DVS

The support from relatives and friends was highlighted in the narratives as being of great importance for the mothers’ and children's escape. Another important source of support is help from social services and the police. This support can take different forms, such as providing support over the phone in an acute situation, helping to find a place at a DVS, or physically helping the mother and children to escape from violence. Amelia described it as follow:

I’d been in contact with the municipality earlier and tried to actualize some form of case, prepare the Soc (social services) for “Hello, you will have a lot to do because I will leave this man and there are a lot of children in the picture.” Thus, I had a contact there, not a person, but I had gotten some contact information and they’d said: “If it becomes necessary, you call [the emergency number]”, and it became necessary that evening, and then I called 112 and said what they’d said I should say… After a while they came out then, Soc… Yes, that's what it looked like. Got help from Soc, who drove me and my child to the Emergency Room to photograph injuries and the police stayed to make sure he [the father] didn’t follow. So, I got help from Soc physically. (Amelia)

Amelia described how social services helped her and her children escape from the violent father, including receiving protection from the police and help in getting the injuries documented, before entering a DVS. A similar scenario was described by Helena:

I called the social services very urgently and asked for help because I didn’t know what to do. I felt really bad and so on. Then they took me very seriously and said, like: “get here at once.” And then we planned a first meeting and made a FREDA assessment, and they made the assessment that we needed sheltered housing, but I didn’t have the children, so it took maybe a day before I got them. He withheld them from me… then he finally agreed to let me meet the children and then it was “run to the car we’re going to social services”… and then he also jumped into the car. So we all went to the social services office where they received us. There were probably five or six [social workers] who sort of stood there and brought me in, and put the father in another room, and took the children away and then they just asked a few questions, then they let me go. And the day before [at the meeting] we’d planned where I would end up, just that I didn’t have the children right then. But then we took it easy there. They [the children] made drawings and we [the mother and the social workers] had a coffee. Then they sent for a taxi, and we left. […] That was probably how we got away anyway. (Helena)

Helena, like Amelia, described how she asked social services for help beforehand. Helena's and Amelia's escape route can thus be understood as planned, even though the acuteness of the situation is clear. Helena's narrative also shows how the safety of the children affected the process of escape and the kind of support received from social services, especially as the father kept them from the mother. This resulted in the mother and children not being able to escape directly, but having to wait until there was a chance for the mother to bring the children to safety. Once they arrived at the social services office, the safety of the children was still important. They were separated from both the mother and the father, and taken care of by social workers, until the mother was given the chance to care for her children. The way in which the children were cared for is also shown in how they were given an opportunity to pause, and do some drawing, together with the mother and social workers, before leaving for a DVS.

In sum, support from family and friends and/or, for example, social services and the police, was given great importance in the mothers’ narratives. The support they were given during the escape affected their children in various ways, from the opportunity to give the children information about what was happening to physically help them flee from a violent situation.

## Discussion

This study investigated the mothers’ experiences of their escape and their considerations regarding their young children's well-being before or during the escape. The escape itself can be understood as one part of the process of leaving a violent situation, or, in other words, what Holmberg and Enander (2005) call the process of breaking up. The focus of our analysis has solely been on the (physical) escape from violence to a DVS, and therefore the process of becoming free and the process of understanding are not discussed here, even though, as Holmberg and Enander (2005) argue, they can be understood as closely linked to the process of breaking up.

In relation to the first research question, the results showed, similar to Vass and Haj-Yahia (2021), that the escape from violence is often sudden, but that there has been some sort of prior planning into how to do so. However, the planning horizon differed in length. For a few, it was an acute situation, or in other words what Fuchs Ebaugh (1988) calls a turning point of an extreme event, from which they had to escape immediately to protect themselves and their children. In line with previous research (Rhodes et al., 2010; Vass & Haj-Yahia, 2021; Zink et al., 2003) and the second research question, our results showed that risks to the health or life of mothers or their children serve as an enabler for leaving the violent relationship. This is also in line with descriptions of the leaving process, and as Holmberg and Enander (2005) show, danger to one's own life or to one's child is often an important turning point for abused women. Furthermore, although the planning horizon for most mothers and their children might be considered short, they sometimes had more time available and manage to use it to their advantage. For example, they could sometimes hide away their own and their children's personal belongings, or contact people in their social network to prepare them. When the time came, they could literally just take the baby stroller and run from their homes to a relative or friend.

In contrast to findings by Holmberg and Enander (2005), our results revealed the importance of social contacts and support at the time of the escape. In the acute cases, the police and other authorities, such as social services, were often involved from the start. In other cases, the mothers turned to people in their personal networks, and these individuals were often of great importance during the escape. They were the ones who initially helped the mothers and children get to safety, or who provided a safe place for them to stay during the first few hours or even weeks. As Fuchs Ebaugh (1988) discusses, the reactions of one's social network are of great importance, especially during the time leading up to a turning point. We agree, because our analysis shows that the period leading up to the escape can be life-threatening, with no possibility of turning back. Accordingly, mothers and children might need physical help during the escape in order to reach safety. In addition, we would like to point out the importance of continued support during the entire leaving process, including the process of becoming free and the process of understanding (Holmberg & Enander, 2005). Even the mother who did not move to a shelter with her children, but instead stayed with a friend, used the social support of a DVS after finding a safe place to live in a new town. The narratives thus highlight the importance of support in its many forms.

Furthermore, it is important to acknowledge the specific situation of children during the escape; however, as our results show, the children were rarely given information about the escape or had time to prepare beforehand. This is also confirmed in studies by Vass and Haj-Yahia (2021) and Øverlien et al. (2009). Moreover, our study shows that children are sometimes left behind due to the circumstances of the escape, and are later picked up by the police or the social services to be rejoined with their mothers. Also, when the mothers and their children stayed with relatives or friends, the mothers had somewhat more time to prepare their children for the move to a DVS and for the changes that would take place. This suggests that the form taken by the escape can impact the stay in secure accommodation. A layover between their home and the shelter or other secure accommodation can, for example, give them some time to reflect on what is happening and plan for the immediate future together with their social network and the authorities, and to be able to prepare their children. Still, not everyone has a social network to call upon for help, and the problem of shelters having to deny mothers and children a safe place due to lack of space, as shown by the report from the National Board of Health and Welfare (2020), can have terrifying consequences.

In conclusion, the escape can be understood as part of a process of leaving violence and, more specifically, an exit process related to various turning points in the mothers’ (and children's) lives. Turning points can be understood as dramatic and sudden events but, as Fuchs Ebaugh (1988) points out, they can also appear gradually. In this study, to answer the research question and in line with Holmberg and Enander (2005), the escape from violence is usually related to an extreme event, often involving danger to the children's health or life. The narratives show, however, that the escape route can differ, in terms of both time and support. Thus, the children's ability to get support from family, friends, or various authorities, whether they have the time to pack items such as their favorite toys, and the extent to which they are informed about what is happening, why they are moving, or what a shelter is, all vary from case to case. As the escape routes from violence to a DVS differ, affecting the situation of the children and their safety, it is important to acknowledge the situation of all children and their different experiences of violence and the escape.

### Limitations

A central limitation was the small sample size, which means that the results should be viewed with caution. A second limitation concerned the lack of an independent coder, which may affect the validity of the study. However, each author did code the material separately to strengthen the quality of the analysis. A third limitation is related to the fact that the children's perspectives on fleeing the violence are told through their mothers, meaning that their experiences are interpreted through another person before they reach the researchers.

### Implications for Theory, Policy, and Practice, and Suggestions for Future Research

Based on these results, it needs to be understood that the escape was just one part of the broader leaving process, which also included the time spent at a shelter or staying somewhere else. The escape affects the choices made by the mothers, and how they interpret what is in the best interests of their children. Therefore, it needs to be part of the understanding of how mothers and children act and react, both at the shelter and afterwards. Regarding the practice, it is thus important that people working with mothers and children who are escaping violence acknowledge that all the processes they go through are interlinked. The effects of the escape continue to manifest at the shelter, or if they are staying somewhere else, and can be understood as part of the leaving process, together with the process of becoming free and the process of understanding. The escape can thus be understood not only as leaving the home where the violence has occurred, but also as part of the mothers’ and children's process of leaving. This is an important point, as it means that the phases created by Rösare (2015) need to be further developed by including the escape as a phase that affects the shelter stay; the shelter stay cannot be understood as something separated from the violence or from the escape and the process of leaving.

The addition of a new phase to Rösare's (2015) original five phases is a theoretical contribution that expands the previous understanding of a shelter stay. More specifically, the escape is understood as a process consisting of several events, which in itself seems to affect the stay at a DVS. This suggests that the process of the escape is important for understanding the experiences of the mothers and their children. Although this is a theoretical contribution to the field, more research is needed to identify mechanisms that positively influence mothers and children who are staying at a DVS. In other words, which events during the process of the escape are important for a positive outcome for the mothers and their children.

**Table 1.** Derivation of Themes and Subthemes from the Analysis.

Main theme	Subtheme	Examples of codes
Timeframe—the planning horizon during the escape	Fleeing in panic	Only the things they had on them at the time. Removal from the home with the help of police or the social services. Couple of days or less. Loss of toys and personal things
	Planned escape	Couple of days or more. Having a plan for where to turn for help and support. Timing the escape when the perpetrator was away. Hiding personal things (essentials). Preparing older children.
Support—informal or formal help during the escape	Relatives and friends as a stopover on the way to a domestic violence shelter	Stopover/a place to emotionally land before moving to the shelter. Temporary solution (if placement at shelter could not be obtained). Start contacting authorities.
	Help from the police and social services when fleeing to a domestic violence shelter	Preparation (that an escape from violence is about to happen). Emergency help. Help with getting a placement at a shelter. Protection.
